# CACUL1/CAC1 Regulates the Antioxidant Response by Stabilizing Nrf2

**DOI:** 10.1038/srep12857

**Published:** 2015-08-04

**Authors:** Yu Kigoshi, Tomomi Fukuda, Tomoyuki Endo, Nami Hayasaka, Shun-ichiro Iemura, Toru Natsume, Fuminori Tsuruta, Tomoki Chiba

**Affiliations:** 1 Graduate School of Life and Environmental Sciences, University of Tsukuba, Tsukuba, Japan; 2 The National Institute of Advanced Industrial Science and Technology, Tokyo, Japan

## Abstract

Nrf2 is the pre-dominant transcription activator responsible for coordinated up-regulation of ARE-driven antioxidant and detoxification genes. The activity of Nrf2 is tightly regulated at basal levels through its ubiquitination by Cul3-Keap1 and consequential degradation. Upon exposure to stress, the Cul3-Keap1 ligase is inhibited, leading to Nrf2 stabilization and activation. Here we describe CACUL1/CAC1 as a positive regulator of the Nrf2 pathway. We found that CACUL1 is up-regulated by Nrf2-activating oxidative stresses in cells and in mice. The association of CACUL1 with the Cul3-Keap1 complex led to a decrease in Nrf2 ubiquitination levels at non-stressed as well as stressed conditions, and sensitized cells for higher Nrf2 activation. Furthermore, CACUL1 knock-down led to a decrease in Nrf2 activity and cell viability under stress. Our results show that CACUL1 is a regulator of Nrf2 ubiquitination, adding another regulatory layer to the Nrf2 antioxidant stress response.

Mammalian cells are often exposed to toxic environmental stresses, many of which disrupt cellular redox state and cause oxidative stress. Oxidative stresses have been implied to cause various diseases such as cancer, diabetes, inflammation, autoimmune diseases, and neurodegenerative diseases. To counterattack the effect of environmental stresses, cells have evolved cytoprotective response systems, which induce the transcription of cytoprotective genes such as antioxidant enzymes and detoxifying enzymes when in need. Nrf2 is the master transcriptional regulator of the cellular redox state, and controls the transcription of its downstream targets by binding onto the antioxidant response element (ARE)^1^,^2^. Many antioxidant genes and phase II detoxifying genes, such as glutathione *S*-transferase (GST), NAD(P)H quinone oxidoreductase (NQO1), and heme oxygenase 1 (HO-1) contain the ARE in their promoter sequences, and are up-regulated by Nrf2 activation.

The Nrf2-mediated antioxidant response is tightly regulated by the Ubiquitin proteasome system. At normal state, Nrf2 is constantly ubiquitinated in the cytoplasm by the Cullin3 (Cul3)-Keap1-Rbx1 Ubiquitin ligase complex, and thereby degraded^3^,^4^,^5^. Keap1, a member of the BTB protein family, acts as the Nrf2 inhibitor by recruiting Nrf2 to the ligase complex, where the Cullin protein Cul3 acts as the scaffold on which the catalytic RING-finger protein Rbx1/Roc1 also assembles onto. Through the dimerization of the BTB domain, Keap1 forms a cherry bob structure, and binds with a single Nrf2 molecule through two motifs: the low affinity DLG and the high affinity ETGE motifs^6^,^7^,^8^. In this way, it is thought that a single Nrf2 molecule is ubiquitinated by two sets of Cul3-Keap1-Rbx1 complexes.

Keap1 is a cysteine rich protein, and upon exposure to electrophiles or reactive oxygen species, the reactive cysteine residues are directly modified by electrophiles, leading to a change in the Keap1 dimer conformation^9^,^10^,^11^,^12^. Consequently, the ETGE motif of Nrf2 remains attached to Keap1 (hinge) while the DLG motif detaches (latch) and prevents Nrf2 ubiquitination in the hinge-and-latch model^11^,^13^,^14^,^15^. The dissociation of Keap1 and Cul3 upon stimulation is also proposed as another model, where the modification of C151 by stress is thought to regulate stress-mediated dissociation of Cul3 and Keap1^16^. In both models, the integrity of the Cul3-Keap1 complex is crucial for effective Nrf2 ubiquitination. Once the Cul3-Keap1 complex is inhibited, Nrf2 accumulates and is able to translocate to the nucleus where it activates ARE-driven gene transcription co-operatively with small Mafs^1^.

The significance of the Nrf2-Keap1 pathway in homeostasis is evident from extensive studies, including those of Nrf2-deficient mice. Nrf2-deficient mice have been shown to have increased sensitivity to toxicants and carcinogens, and are known to develop inflammatory disorders, due to decrease in cellular capacities for ROS elimination^17^,^18^,^19^,^20^,^21^,^22^,^23^,^24^. Increase in Nrf2 levels are seen in various forms of cancer due to changes in its regulation such as transcription, and mutations in Keap1 or Nrf2 themselves^25^,^26^,^27^,^28^,^29^,^30^,^31^,^32^. Such dysregulation of Nrf2 in cancer cells leads to high activity of target genes, resulting in resistance to chemopreventive drugs in some cancers. Therefore detailed understanding of the regulation of this system is of high importance.

CACUL1/CAC1 is a presumptive Cullin domain containing protein whose function is yet to be characterized in depth. Several recent studies have shown that CACUL1 is highly expressed in various cancers, and promotes cell cycle progression in some cancer cell lines^33^,^34^,^35^. It has also been reported that CACUL1 is involved in ERα and RARα regulation by binding to and repressing their transcription activity in cells^36^,^37^. Moreover CACUL1 has been reported to be down regulated in hippocampus of Alzheimer disease patients, and up-regulated by H_2_O_2_ and Aβ in SHSY-5Y cells^38^. It was also shown that CACUL1 knock-down leads to increase in oxidative stress, suggesting a role in cellular stress responses^38^. However, its biochemical function and biological relevance in stress response mechanisms is not fully understood.

In this study, we show that CACUL1 is a stress-induced protein that associates with the Cul3-Keap1-Nrf2 complex, and negatively regulates Nrf2 ubiquitination. Under oxidative stress, CACUL1 sensitized cells for higher Nrf2 activation. Furthermore, CACUL1 knock-down led to lower Nrf2 activity and lower cell viability under stressed condition. These data suggest that CACUL1 is a novel positive regulator of Nrf2 and the antioxidant stress response, involved in cell survival under oxidative stress.

## Results

### CACUL1 is a stress-induced protein

**Figure 1:**
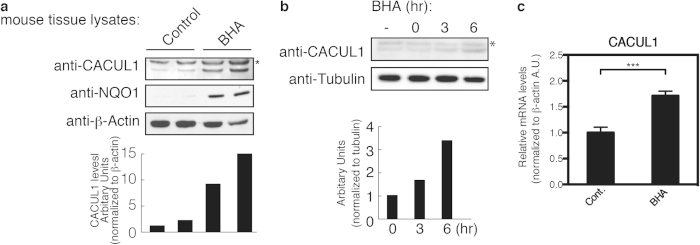
CACUL1 is up-regulated by stresses. (**a**) Kidneys of C57BL/6 female mice fed with BHA for 14 days, were lysed and detected for CACUL1, NQO1 and β-Actin protein levels. Data for 2 individual mice are shown for control and BHA fed animals each. Asterisk indicates nonspecific bands. The relative CACUL1 proteins levels were quantified using ImageJ software. (**b**) HeLa cells were treated with 250 μM BHA for the indicated times. The cell lysates were immunoblotted for CACUL1 and Tubulin, and the images were quantified using ImageJ software. Asterisk indicates nonspecific bands. (**c**) Total RNAs from HEK293 cells treated with 250 μM BHA for 6 hrs were purified, and reverse transcribed. The cDNA was used in real-time qPCR assays for CACUL1, and β-Actin as control. Mean and s.d. from a representative experiment performed in triplicate is shown. ***P ≤ 0.001 using one-way ANOVA. Un-cropped images with molecular weights are shown in Supplementary Figure S7.

As CACUL1 was reported to be up-regulated by H_2_O_2_ in cells^38^, we speculated that CACUL1 may be related to the Nrf2 pathway. Therefore we asked if CACUL1 can be up-regulated in mice by butylated hydroxyanisole (BHA), an oxidative stress inducer (Supplementary Figure S1) that activates the Nrf2 pathway. Mice were fed with BHA for 14 days, and changes in protein expression were analyzed in tissue lysates (Fig. 1a). Compared to controls, the protein levels of CACUL1 and NQO1 were substantially increased in the kidneys of BHA-fed mice, but not in other tissues such as liver (Supplementary Figure S2), suggesting that CACUL1 protein levels are up-regulated under Nrf2 activating stress in a tissue specific manner. We next investigated CACUL1 protein and mRNA levels in BHA treated cells. When HeLa cells were treated with BHA, an increase in CACUL1 protein levels were seen in a time-dependent manner (Fig. 1b). Increase in CACUL1 mRNA levels was also seen in cells treated with BHA (Fig. 1c). These data indicate that CACUL1 is a target gene up-regulated by oxidative stresses, suggesting a link between CACUL1 expression and the Nrf2 pathway.

### CACUL1 associates with the Cul3-Keap1 complex

**Figure 2:**
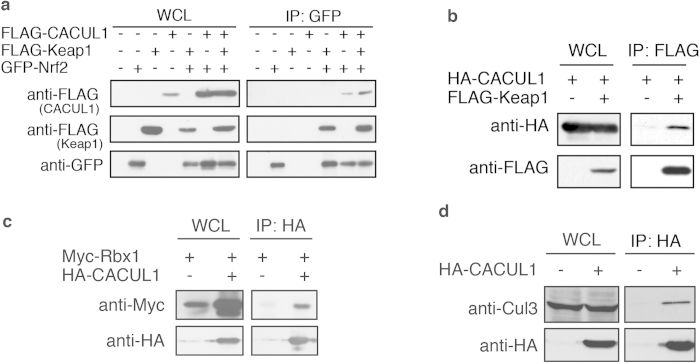
CACUL1 associates with Cul3-Keap1 ligase components. (**a**–**d**) HEK293T cells were transfected with GFP-Nrf2, FLAG-Keap1, or FLAG-CACUL1 (**a**), FLAG-Keap1 and HA-CACUL1 (**b**), Myc-Rbx1 and HA-CACUL1 (**c**), and HA-CACUL1 (**d**) as indicated. Cell lysates were immunoprecipitated with anti-GFP (**a**), anti-FLAG (**b**) or anti-HA (**c** and **d**) antibodies and immunoblotted as indicated. Un-cropped images with molecular weights are shown in Supplementary Figure S7.

CACUL1 contains a presumptive Cullin domain, a domain largely associated with the Ubiquitin system. Furthermore, CACUL1 was found to interact with BTB protein by mass spectrometric analysis (Supplementary Table 1). Therefore we speculated that CACUL1 might be associated in Nrf2 regulation through the Cul3-Keap1 ligase complex. We first investigated CACUL1 binding with Nrf2 by immunoprecipitation experiment. Cells were transfected with GFP-tagged Nrf2, FLAG-tagged CACUL1 and Keap1, and were immunoprecipitated with anti-GFP antibody. A slight amount of CACUL1 was co-precipitated with Nrf2, which increased with Keap1 co-expression (Fig. 2a), suggesting that CACUL1 interacts with Nrf2 through Keap1. To investigate if CACUL1 binds with the Cul3-Keap1 complex, we further assessed CACUL1 binding with Keap1, Cul3 and Rbx1, the components of the Ubiquitin ligase responsible for Nrf2 ubiquitination. Immunoprecipitation of cell lysates expressing FLAG-tagged Keap1 and HA-tagged CACUL1 using anti-FLAG antibody, resulted in CACUL1 co-precipitation indicating that CACUL1 does indeed interact with Keap1 (Fig. 2b). CACUL1 interaction with Rbx1 was also detected in immunoprecipitation of Myc-tagged Rbx1 with HA-tagged CACUL1 (Fig. 2c). Furthermore, endogenous Cul3 co-precipitated with CACUL1, suggesting that CACUL1 associates with the Cul3-Keap1 complex (Fig. 2d). Altogether, these data indicate that CACUL1 associates with the Cul3-Keap1-Nrf2 complex.

### CACUL1 attenuates Nrf2 ubiquitination

**Figure 3:**
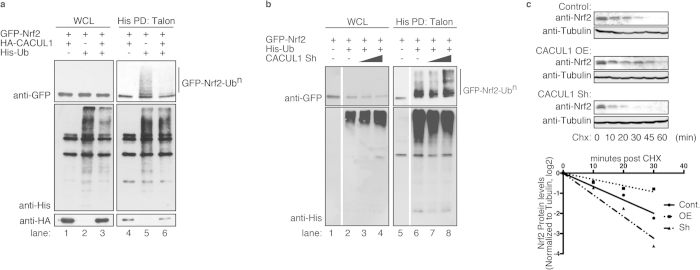
CACUL1 stabilizes Nrf2 by attenuating Nrf2 ubiquitination. (**a**,**b**) HEK293T cells transfected with GFP-Nrf2 with His-Ub and HA-CACUL1 (**a**) or pSIREN-CACUL1 (**b**) were treated with 20 μM MG132 for 30 min, lysed, and ubiquitinated proteins were pulled down with Talon metal affinity resin in denatured conditions. The samples were then immunoblotted as indicated. (**c**) HEK293T cells transfected with empty vector, FLAG-CACUL1, or pSIREN-CACUL1 in single dishes, were re-plated onto 6 well plates. Prior to harvest, cells were treated with 50 μg/ml CHX for the indicated times, and cell lysates were immunoblotted for endogenous Nrf2 and Tubulin as control. Band intensities were quantified using ImageJ software, and relative Nrf2 levels normalized to Tubulin were calculated and plotted. Un-cropped images with molecular weights are shown in Supplementary Figure S7.

As CACUL1 interacted with the Cul3-Keap1 complex, we next investigated the effect of CACUL1 on Nrf2 ubiquitination and stability. First, Nrf2 ubiquitination was verified by the His-Ubiquitin pull down assay in the cell under proteasomal inhibition. Cells were transfected with His-tagged Ubiquitin and Nrf2, treated with MG132, and denatured. The denatured lysates were then applied onto Talon-beads to pull down ubiquitinated proteins. In the control sample (Fig. 3a, lane 5), His-Ubiquitin conjugated Nrf2 was observed as a smear, reflecting the variety of Ubiquitin chains conjugated onto Nrf2. Upon CACUL1 expression, this smear of ubiquitinated Nrf2 drastically decreased in comparison to control (lane 6), suggesting that CACUL1 attenuates Nrf2 ubiquitination. When CACUL1 was knocked-down (Supplementary Figure S3), the levels of ubiquitinated Nrf2 increased (Fig. 3b, lane 7 and 8) in comparison to control (lane 6), suggesting that CACUL1 attenuates Nrf2 ubiquitination. As CACUL1 inhibited Nrf2 ubiquitination, we also asked whether CACUL1 affects the formation of Cul3-Keap1-Nrf2 complex. CACUL1 expression had little effect on Nrf2-Keap1 interaction as well as Keap1-Cul3 interaction (Supplementary Figure S4a), suggesting that CACUL1 does not affect Cul3-Keap1-Nrf2 complex formation. Furthermore, when we investigated the effect of CACUL1 on Cul3-associated ubiquitinated proteins, it decreased with CACUL1 expression, suggesting that CACUL1 decreases the ligase activity of the Cul3 complex (Supplementary Figure S4b).

Next, we investigated Nrf2 stability in a cycloheximide chase experiment. CACUL1 over-expressed or knock-down cells were subjected to cycloheximide treatment for indicated times, and cell lysates were immunoblotted to detect endogenous Nrf2 (Fig. 3c). In control samples, Nrf2 was rapidly degraded with a half-life of approximately 15 minutes in agreement with previous studies^39^. When CACUL1 was over-expressed, Nrf2 stabilized and its half-life lengthened to about 30 minutes, while CACUL1 knock-down led to destabilization of Nrf2 shortening its half-life to about 10 minutes. These results indicate that CACUL1 stabilizes Nrf2 by regulating Nrf2 ubiquitination.

### CACUL1 affects Nrf2 activity under stress, but not at basal condition

**Figure 4:**
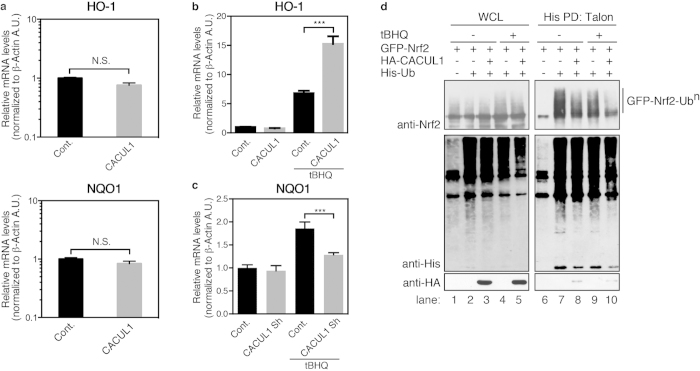
CACUL1 affects Nrf2 activity under stress, but not at basal conditions. (**a**,**b**,**c**) Total RNAs from CACUL1 over-expressing (**a** and **b**) or knocked-down (**c**) cells were purified, and reverse transcribed. Cells were treated without (**a**) or with (**b** and **c**) 50 μM tBHQ for 4 hrs prior to harvest. The cDNA was used in real-time qPCR assays for NQO1, HO-1, and β-Actin as control. Mean and s.d. from a representative experiments performed in triplicate are shown. N.S.: not significant, ***P ≤ 0.001, using one-way ANOVA. (**d**) HEK293T cells transfected with GFP-Nrf2, His-Ub and HA-CACUL1 as indicated were treated with 10 μM MG132 with or without 50 μM tBHQ for 4 hrs, lysed, and ubiquitinated proteins were pulled down with Talon metal affinity resin in denatured conditions. The samples were then immunoblotted as indicated. Un-cropped images with molecular weights are shown in Supplementary Figure S7.

We next investigated whether CACUL1 affects Nrf2 activity and localization. First, to ask if CACUL1 affects Nrf2 activity, the mRNA levels of the Nrf2 target genes NQO1 and HO-1 were investigated at basal conditions. In CACUL1 over-expressed cells, the transcripts of NQO1 and HO-1 were not significantly up-regulated (Fig. 4a), indicating that though CACUL1 stabilizes Nrf2, it is not sufficient to enhance Nrf2 activation. The subcellular distribution of Nrf2 was also not drastically affected with CACUL1 over-expression or knock-down (Supplementary Figure S5a), indicating that Nrf2 stabilization by CACUL1 does not lead to changes in its subcellular localization.

We next investigated the effect of CACUL1 under stressed conditions. When the mRNA levels of Nrf2 target genes were tested under stress, it significantly increased with CACUL1 over-expression (Fig. 4b), suggesting that CACUL1 sensitizes cells for effective Nrf2 activation under stressed conditions. When CACUL1 was knocked-down, target gene transcription levels did not significantly change at basal conditions (Fig. 4c). However, when treated with the oxidative stress inducer tBHQ that stabilizes Nrf2 (Supplementary Figure S5c), NQO1 mRNA levels were significantly increased in control cells. In contrast, the induction of NQO1 was lower in CACUL1 knock-down cells, suggesting that CACUL1 is important for Nrf2 activity under stress. We also investigated Nrf2 subcellular localization under stress. When treated with tBHQ and BHA, the Nrf2 localizations changed from cytoplasmic and nuclear in the control cells (Supplementary Figure S5a and d, upper panels) to mainly nuclear (Supplementary Figure S5b, upper panel and d, lower panel), indicating that these stresses induce Nrf2 translocation. Nuclear translocation of Nrf2 were similarly induced by stress in CACUL1 over-expression or knock-down cells (Supplementary Figure S5b), suggesting that though CACUL1 regulates Nrf2 activity under stressed conditions, it does not regulate Nrf2 localization.

As CACUL1 expression sensitized cells for higher Nrf2 activation under stress, we next performed His-Ubiquitin pull down assay to see the effect of CACUL1 on Nrf2 ubiquitination under proteasomal inhibition, in cells treated with stress. When the cells were treated without or with tBHQ in addition to MG132, slight Nrf2 stabilization was observed in the lysate samples of tBHQ treated cells (Fig. 4d, lanes 4 and 5). In the pull down samples, a decrease in the amount of ubiquitinated Nrf2 was observed in tBHQ treated samples (lanes 9 and 10), indicating that Nrf2 ubiquitination is inhibited by tBHQ treatment leading to its stabilization. When CACUL1 was co-expressed, a further decrease in the amounts of ubiquitinated Nrf2 was observed (lane 10), suggesting that CACUL1 reduces Nrf2 ubiquitination even under stressed condition. Altogether, these data indicate that CACUL1 regulates Nrf2 ubiquitination during stressed conditions, leading to enhancement in Nrf2 activation.

### CACUL1 is important for cell survival

**Figure 5:**
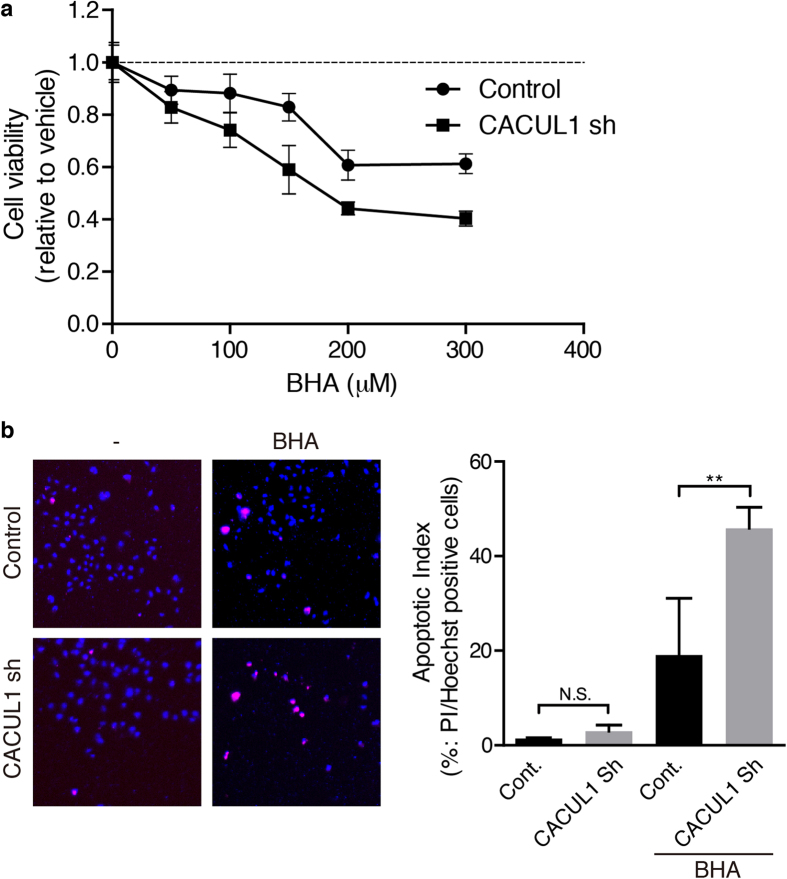
CACUL1 knock-down leads to cell vulnerability. (**a**) HEK293 cells transfected with pSIREN-CACUL1 or control vectors were treated with indicated amounts of BHA for 24 hrs, and cell viability assay was performed. Mean ± s.d. from a representative experiment performed in triplicate is indicated. (**b**) Cells transfected with pSIREN(-DsRed)-CACUL1 or control vectors were treated with 300 μM of BHA for 3 hrs, and stained with PI and Hoechst 33342 prior to fixation. Fixed cells were then observed, and the percentage of co-staining cells was quantified. Mean and s.d. from a representative experiment performed in triplicate. N.S.: not significant, **P ≤ 0.01, using one-way ANOVA.

To investigate CACUL1 function in cell survival, we next performed cell viability assays using CACUL1 knock-down cells (Fig. 5a). When control or CACUL1 knock-down cells were treated with indicated amounts of BHA for 24 hours, cell viability decreased in proportion to BHA concentration, which further decreased in CACUL1 knock-down cells, indicating that CACUL1 knock-down leads to higher vulnerability to stress. We next checked for cell death by the PI/Hoechst double staining assay. HeLa cells transfected with CACUL1 knock-down or control constructs were treated with BHA and observed for apoptotic cells. In comparison to control cells, more apoptotic cells were observed in CACUL1 knock-down cells (Fig. 5b). These data suggest that the decrease in cell viability of CACUL1 knock-down cells is due to increased apoptosis. The vulnerability of these cells may be partly due to lower Nrf2 activity in CACUL1 knock-down cells under stress, as seen in Fig. 4c. Altogether, these data indicate that CACUL1 is important for cell survival under stress.

## Discussion

Here we show that the stress-induced protein CACUL1 is a novel factor that regulates Nrf2 activity under stress. CACUL1 associated with the Cul3-Keap1 complex, and attenuated the ubiquitination of Nrf2, both during stressed and non-stressed conditions in the cell, thus regulating Nrf2 stability. During stress, Nrf2 activity increased with CACUL1 over-expression, and decreased with CACUL1 knock-down. However, CACUL1 did not affect Nrf2 activity at non-stressed condition, suggesting that Nrf2 stabilization by CACUL1 is not sufficient to promote Nrf2 activation, and mechanisms other than Nrf2 stabilization may be involved in this regulation. It has been suggested that the counter balance of multiple nuclear localization signals in Nrf2 are important to regulate its subcellular localization, and that the inactivation of the redox sensitive NES_TA_, a NES in the transactivation domain of Nrf2, may be an important factor in regulating Nrf2 translocation^40^,^41^. This suggests a self-sufficient mechanism for which Nrf2 itself is able to sense stresses and translocate to the nucleus. Such Ubiquitin independent activation mechanisms may explain why Nrf2 stabilization by CACUL1 alone did not lead to Nrf2 translocation and activation.

Studies suggest that Keap1 re-targets nuclear Nrf2 for degradation by exporting Nrf2 to the cytoplasm after stress response^42^. CACUL1 may also play a role in this pathway. By attenuating the ubiquitination of Nrf2 that is exported out of the nucleus by Keap1, it is possible that CACUL1 prevents excessive degradation to maintain an adequate level of Nrf2 during and after the post-induction repression process. This may in part explain the additional attenuation of Nrf2 ubiquitination seen with CACUL1 expression under stress.

In CACUL1 knock-down cells, induction of Nrf2 target genes was attenuated in comparison to control cells, and these cells were less viable indicating that Nrf2 stabilization by CACUL1 is an important mechanism for cell survival under stress. Several studies have shown that cellular defense can be enhanced by stress pre-treatment known as the electrophile counter attack response^43^. As Nrf2 plays a key role in pre-treatment enhancement of cell protection against stronger second-round attacks^44^,^45^, CACUL1 may also contribute to this model. The facts that CACUL1 stabilizes Nrf2, and is up-regulated by Nrf2 activating stresses, suggest a role for CACUL1 in some feedback mechanism. Induction of CACUL1 can be expected to help further stabilization of Nrf2 during and after stress induction. This may induce prolongation of the Nrf2 response, or secure higher amounts of Nrf2 pooled in the cells after initial stress, allowing a faster response for second bouts or longer-termed stress. In support of this idea, our data show that CACUL1 over-expression further decreases the ubiquitination levels of Nrf2, and induces higher mRNA levels of Nrf2 target gene HO-1 under tBHQ stress (Fig. 4b,d).

In BHA feeding experiments in mice, CACUL1 up-regulation was seen in the kidney (Fig. 1a), but not so evident in other tissues such as the liver (Supplementary Figure S2), suggesting that CACUL1 induction by stress varies by tissue type. Moreover, CACUL1 is expressed in a variety of tissues at basal level (Supplementary Figure S6), but not in all tissues examined. Furthermore, CACUL1 expression was higher in tissues that expressed high levels of Nrf2. This difference in CACUL1 expression between tissues at non-stressed and stressed conditions may contribute in some extent to the difference in stress responses among tissues. Moreover, these observations poses the interesting idea that the effects or regulation of CACUL1 differ depending on the cell types.

High oxidation levels, as well as defects that lead to high Nrf2 levels, are known characteristics of various cancer cell lines. Moreover, high CACUL1 levels in cancer cells have been reported in previous studies^33^,^34^,^35^. Our data concur with these observations, as high CACUL1 levels in cancers may lead to higher Nrf2 stabilization and activity in these cells, which can be expected to contribute to cellular tolerance against stress and cell death. Indeed, CACUL1 is reported to inhibit cisplatin induced apoptosis in gastric cancer cell lines^33^. Nrf2 stabilization may contribute to the cell cycle progression by CACUL1, as Nrf2 has been shown to be important for cell cycle progression in cancers^46^,^47^,^48^. Overall, CACUL1 may be a potential target for cancer treatment.

In conclusion, we have shown that CACUL1, a stress induced protein, plays a positive role in Nrf2 regulation by attenuating Nrf2 ubiquitination, thereby enhancing cell survival under oxidative stress. The importance of the Nrf2 pathway *in vivo* is supported by extensive studies showing its relation to various diseases such as cancers. Furthermore, Nrf2 activity has been implicated in protection against neurodegenerative diseases^49^, and it has been reported that CACUL1 knock-down in the neuroblastoma SHSY-5Y cell line leads to increase in oxidative stress^38^. By playing a role in fine-tuning the regulation of Nrf2, CACUL1 may contribute to the pathological conditions of these disorders.

## Materials and Methods

### Expression Plasmids

Epitope-tagged full length CACUL1 was amplified from human placenta cDNA library using Phusion High Fidelity DNA polymerase (Thermo) and cloned into pcDNA and pcDEF^50^ plasmids. The constructs, pCAGEN-His-Ub from Y. Gotoh (University of Tokyo, Japan), pcDNA-EGFP-C4-Nrf2 obtained from Addgene (plasmid 21549), pcDNA-Ub, pcDNA-Myc-Rbx1, pcDNA-FLAG-Cul3, and pCMV-Keap1 have been described previously^4^,^29^,^51^,^52^,^53^,^54^,^55^. For the sh CACUL1 knock-down plasmid, the following oligo sequences, Top: 5′-GATCCGGGGTGATCATGATGTTGAAGATTCAAGAGATCTTCAACATCATGATCACCCTTTTTTACGCGTG-3′, Bottom: 5′-AATTCACGCGTAAAAAAGGATGGTGCCATAGATCAACTTCTCTTGAAAGTTGATCTATGGCACCATCCCG-3′, were inserted into the pSIREN-DNR-DsRed-Express (CLONTECH) to target the sequence 5′-GGATGGTGCCATAGATCAACT-3′ (Supplementary Figure S3). For the pSIREN(-DsRed) constructs, the Ds-Red was omitted from the pSIREN vector by *Nco I* digestion.

### Antibodies

The following antibodies were used for immunoblot analyses: anti-c-Myc (Santa Cruz, 9E10), anti-FLAG (Sigma, M2), anti-HA (Santa Cruz, Y11, Roche, 3F10), anti-His (GE healthcare), anti-Nrf2 (Santa Cruz, H300), anti-α-Tubulin (Sigma, DM1A), anti-GFP (MBL, 598), anti-NQO1 (Abcam, A180), and anti-β-Actin (Cell Signaling, D6A8). Anti-CACUL1 antibody was raised by rabbit immunization against recombinant fragment (a.a. 118 ~ 369) of human CACUL1. Anti-Cul3 antibody has been described previously^53^. Peroxidase-conjugated anti-rabbit, anti-rat (Jackson ImmunoResearch) and anti-mouse antibodies (Jackson ImmunoResearch, Sigma) were used as secondary antibodies. For immunofluorescence analyses, anti-Nrf2 (Abcam, EP1808Y) and anti-FLAG (Sigma, M2) were used with Alexa Fluor 488-, and Alexa Fluor 594-conjugated anti-mouse and anti-rabbit antibodies (Life Technologies).

### Cell Culture and Transfection

HEK293T, HEK293, and HeLa cells were cultured in Dulbecco’s modified Eagle’s medium (low glucose) (Wako) supplemented with 10% fetal bovine serum, and 1% penicillin/streptomycin (Invitrogen) in a 37 °C incubator with 5% CO_2_. Transfections were carried out using FuGENE 6 transfection reagent (Roche Applied Science) or polyethylenimine (Polyscience, Inc.) according to the manufacturers’ specifications.

### Reagents

MG132 (benzyloxycarbonyl-Leu-Leu-Leu-H) (Peptide Institute) was used for proteasome inhibition; cycloheximide (Wako) for translation inhibition; *tert*-butylhydroquinone (tBHQ) (Wako) and butylated hydroxyanisole (BHA) (Sigma) for stress induction. CellROX Green reagent (Life Technologies) was used for cellular ROS detection.

### Immunoprecipitation and Immunoblot Analyses

Cells were lysed with ice-cold lysis buffer (20 mM Tris-HCl, pH 7.5, 150 mM NaCl, 1 mM EDTA, 1 mM dithiothreitol (DTT) with 0.5% NP-40 or CHAPS). The cleared supernatant was subjected to immunoprecipitation with anti-FLAG M2-agarose (Sigma), anti-HA agarose (Sigma), or anti-GFP (MBL) antibody with protein G agarose beads (Thermo Scientific). The beads were washed with lysis buffer after incubation at 4 °C overnight. The samples were then boiled in SDS sample buffer, separated by SDS-PAGE, and immunoblotted using Western Lightning Plus-ECL reagent (PerkinElmer Life Sciences).

### His Pull-Down Assay

Cells were transfected with the indicated constructs and lysed in extraction buffer (6 M guanidinium-HCl, 50 mM sodium phosphate buffer (pH 8.0), 300 mM NaCl and 5 mM imidazole). Cell lysates were sonicated briefly, centrifuged, and the cleared supernatants were incubated with Talon metal affinity resin (Clontech) at 4 C° overnight. The resin were washed with wash buffer (50 mM sodium phosphate buffer (pH 8.0), 300 mM NaCl and 5 mM imidazole) and subjected to immunoblot analyses^56^.

### Immunofluorescence Analyses

HeLa cells were cultured on coverslips and transfected with various expression plasmids. The cells were fixed in 4% paraformaldehyde in PBS for 10 min, permeabilized and blocked in 0.5% Triton X-100, 5% BSA in PBS for 30 min. The coverslips were then immersed in primary antibodies followed by secondary immunofluorescent antibodies diluted in 5% BSA in PBS. Nuclei were stained with Hoechst 33342 (Life Technologies). The coverslips were mounted onto slides using Fluoromount/Plus mounting solution (Diagnostic BioSystems), and images were obtained using a fluorescence microscope (Keyence model BZ-9000).

### RNA Extraction, Reverse Transcription and qPCR Analyses

Total RNAs were extracted using RNeasy mini kit (Qiagen), and mRNAs were reverse transcribed using Super Script III First-strand synthesis system (Invitrogen) according to the manufacturers’ instructions. qPCR analyses were performed using the Thunderbird Sybr qPCR Mix (TOYOBO) on the Thermal Cycler Dice Real-Time System II (Takara, TP800). The following primers were used in the reactions:

CACUL1 forward: TGGGTTCAGA TGGCTCCAAC TCTATTTTC

CACUL1 reverse: GACTGGTCAC CCCTTGTAAA ACCATTCTG

β-Actin forward: TGGACATCCGCAAAGACCTG

β-Actin reverse: GGAGGAGCAATGATCTTGATCTTC

HO-1 forward: GGAACTTTCAGAAGGGCCAGGT

HO-1 reverse: GACTGGGCTCTCCTTGTTGC

NQO1 forward: GGGATCCACGGGGACATGAATG

NQO1 reverse: GGATTTGAATTCGGGCGTCTGCTG

### Data Analyses

Data for qPCR were analyzed using the Thermal Cycler Dice Real Time System software (Takara), using the ΔΔCt method on Ct (Crossing Point). The significance of data was calculated, using one-way ANOVA and Tukey multiple comparison tests, and graphs were drawn using Prism software (GraphPad Software).

### Mouse Feeding Experiments

Female C57BL/6 Mice of 10 ~ 12 weeks of age were fed *ad libitum* on MF powder feed (Oriental Yeast) with free access to water. Mice were fed with or without 0.5% (w/w) BHA for 14 to 15 days. All animal experiments were approved by and performed in accordance with the guidelines of the University of Tsukuba’s Regulation of Animal Experiments Committee.

### Cell Viability Assay

Cells transfected with control or pSIREN-CACUL1 plasmids were plated onto 96 well plates, and treated with BHA for 24 hours. Cell viability was measured using Cell Counting Kit-8 (Dojindo) according to the manufacture’s instructions.

### PI/Hoechst Double Staining Assay

Cells transfected with pSIREN(-DsRed) control or CACUL1 knock-down plasmids were plated onto coverslips. Cells were treated with 300 μM of BHA for 2.5 hours, and further stained with PI solution (Dojindo) and Hoechst 33342 (Life Technologies) for 30 min prior to fixation, and observed under a fluorescent microscope.

## Additional Information

**How to cite this article**: Kigoshi, Y. *et al.* CACUL1/CAC1 Regulates the Antioxidant Response by Stabilizing Nrf2. *Sci. Rep.* **5**, 12857; doi: 10.1038/srep12857 (2015).

## Supplementary Material

Supplementary Information
