# Supplementary material for: CACUL1/CAC1 Regulates the Antioxidant Response by Stabilizing Nrf2
Source: Sci Rep. 2015 Aug 4;5:12857. doi: 10.1038/srep12857 (PMC4523873; doi:10.1038/srep12857)
Supplement: Supplementary Information [file srep12857-s1.pdf]

Supplementary Information for

CACUL1/CAC1 Regulates the Antioxidant Response by Stabilizing Nrf2

Yu Kigoshi[1], Tomomi Fukuda[1], Tomoyuki Endo[1], Nami Hayasaka[1], Shun-ichiro Iemura[2], Toru Natsume[2], Fuminori Tsuruta[1], Tomoki Chiba[1,*]

1: Graduate School of Life and Environmental Sciences, University of Tsukuba, Tsukuba, Japan
2: The National Institute of Advanced Industrial Science and Technology, Tokyo, Japan
*: To whom correspondence should be addressed
E-mail: tchiba@biol.tsukuba.ac.jp

Supplementary Table 1. Proteins identified by mass spectrometry of CACUL1 immunoprecipitants.

Gene Symbol	Score	Protein Name/Description
CCDC88A	3	coiled-coil domain containing 88A
KLHL7	4	kelch-like family member 7 (BTB protein family)
KPNA1IKPNA5IKPNA6	4	karyopherin alpha 1 (importin alpha 5)Ikaryopherin alpha 5 (importin alpha 6)Ikaryopherin alpha 6 (importin alpha 7)
KPNA6	4	karyopherin alpha 6 (importin alpha 7)
SNX4	3	sorting nexin 4

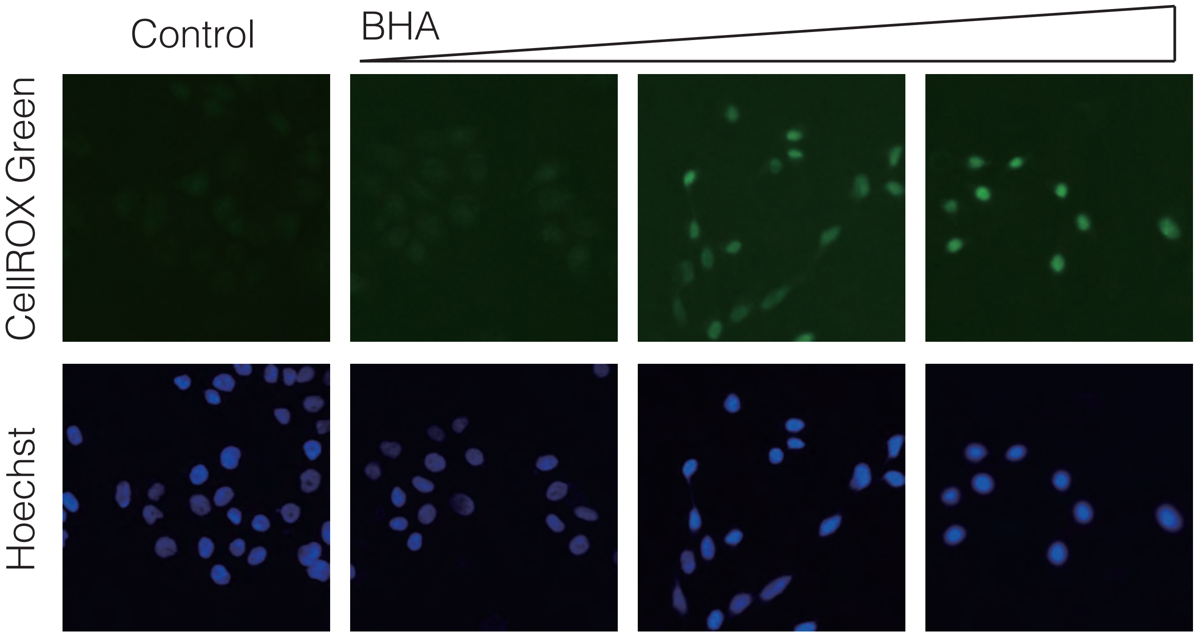

Supplementary Figure S1. Cellular ROS accumulation with BHA treatment.

HeLa cells were treated with DMSO, 250 μM, 500 μM and 750 μM BHA for 4 hours. Then cells were incubated with CellROX Green reagent and Hoechst 33342 for 30 mins, fixed, and observed under the fluorescent microscope.

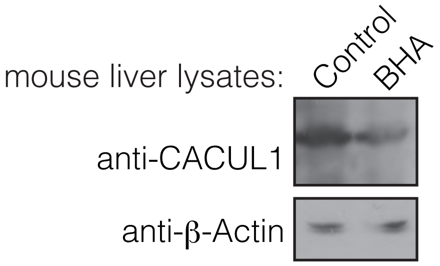

Supplementary Figure S2. CACUL1 is not greatly induced by BHA in mice liver.

Livers of C57BL/6 female mice fed with BHA for 14 days were lysed and detected for CACUL1 and β-Actin protein levels.

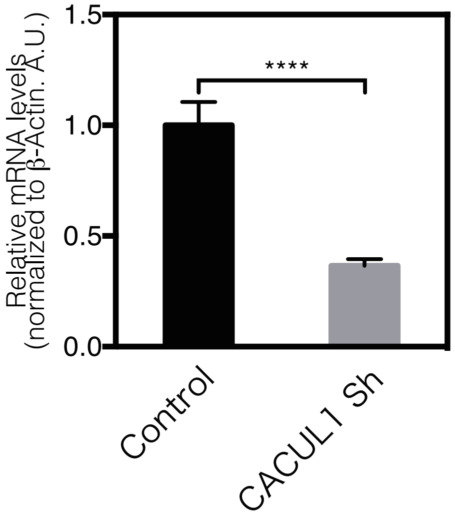

Supplementary Figure S3. Knock-down of CACUL1.

Total RNAs from HeLa cells transfected with Sh CACUL1 plasmid were purified, and reverse transcribed. The cDNA was used in real-time PCR assays for CACUL1 and β-Actin as control. Mean and s.d. of an experiment performed in quadruplicate, ****$P \leq 0.0001$, using two-tailed student' s t-test.

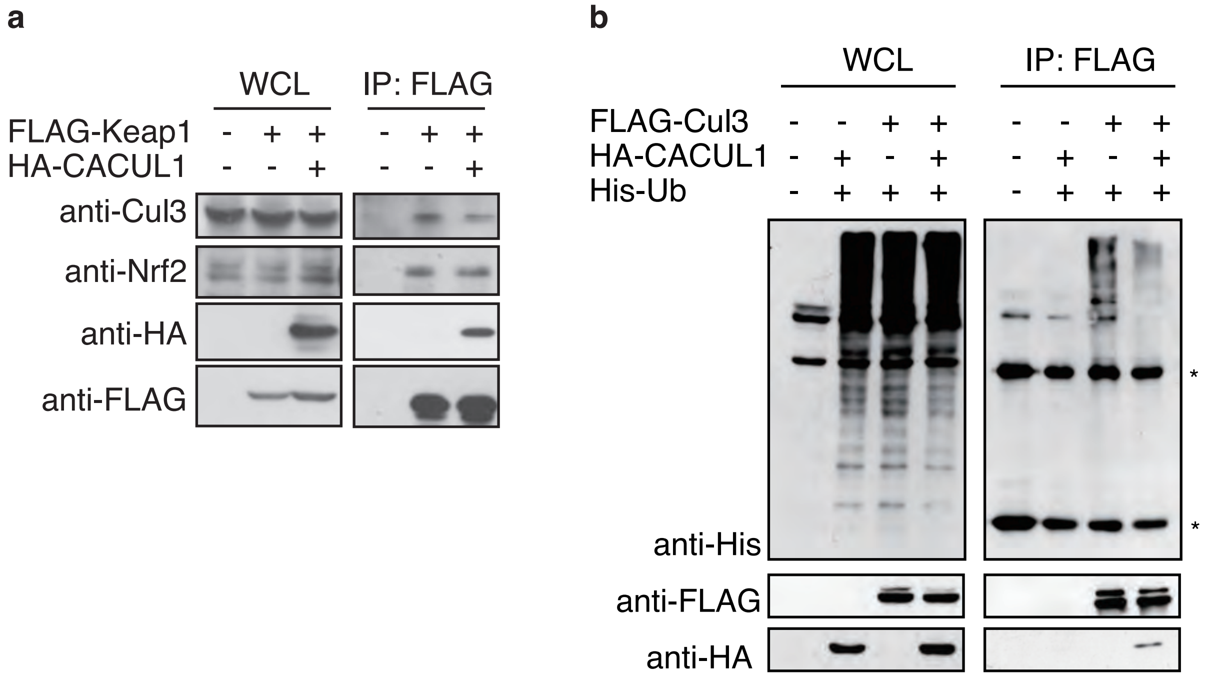

Supplementary Figure S4. CACUL1 does not affect Cul3-Keap1-Nrf2 binding, and attenuates Cul3 binding with ubiquitinated proteins.

a. Cells were transfected with FLAG-Keap1 and HA-CACUL1 and cell lysates were immunoprecipitated with anti-FLAG antibody and detected for Cul3-Keap1-Nrf2 complex components as indicated. **b.** HEK293T cells transfected with FLAG-Cul3, HA-CACUL1, and His-Ub were treated with 20 μM MG132 for 1 hr prior to cell lysis. Cell lysates were immunoprecipitated with anti-FLAG antibody and immunoblotted as indicated. Asterisks indicate IgG bands.

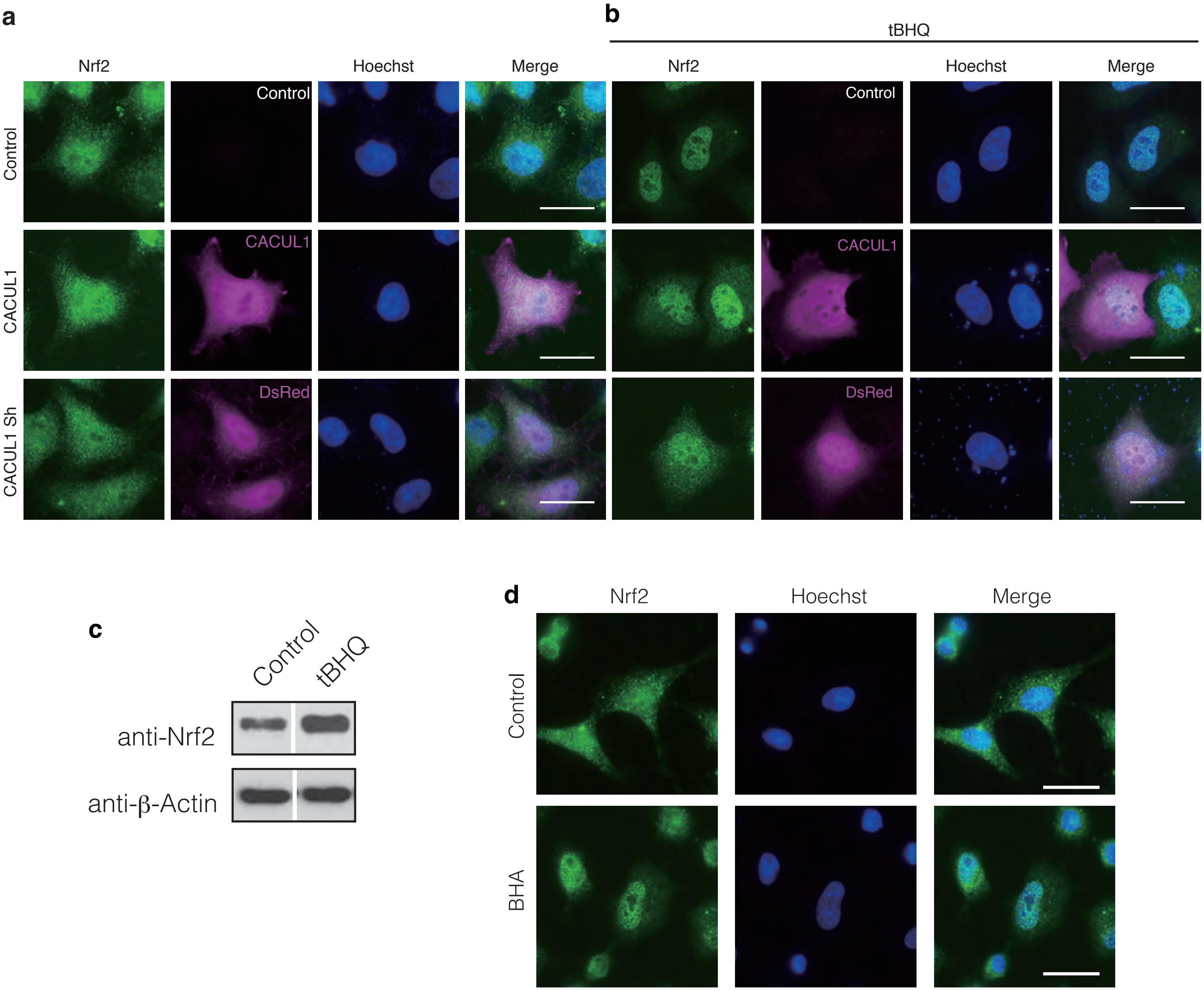

Supplementary Figure S5. CACUL1 does not drastically affect Nrf2 localization.

a, b. HeLa cells transfected with FLAG-CACUL1 or pSIREN-CACUL1 were treated without (a) or with (b) 50 μM tBHQ for 1 hr, fixed and stained using anti-FLAG and anti-Nrf2 antibodies to detect exogenous CACUL1 and endogenous Nrf2. Knock-down cells were detected by DsRed fluorescence. Nuclei were stained using Hoechst 33342. Scale bars indicate 20 μm.**c.** HEK293T cells were treated without or with 50 μM tBHQ for 4 hrs. Cells were lysed and blotted for Nrf2 and β-Actin. **d.** HeLa cells were treated with 250 μM BHA for 1 hr, fixed and stained using anti-Nrf2 antibody. Nuclei were stained using Hoechst 33342. Scale bars indicate 20 μm.

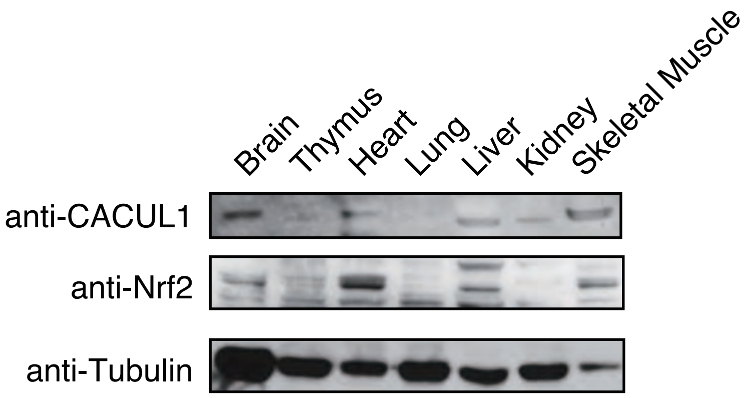

Supplementary Figure S6. CACUL1 is expressed in multiple tissues.

Murine tissue lysates were immunoblotted for CACUL1, Nrf2 and Tubulin.

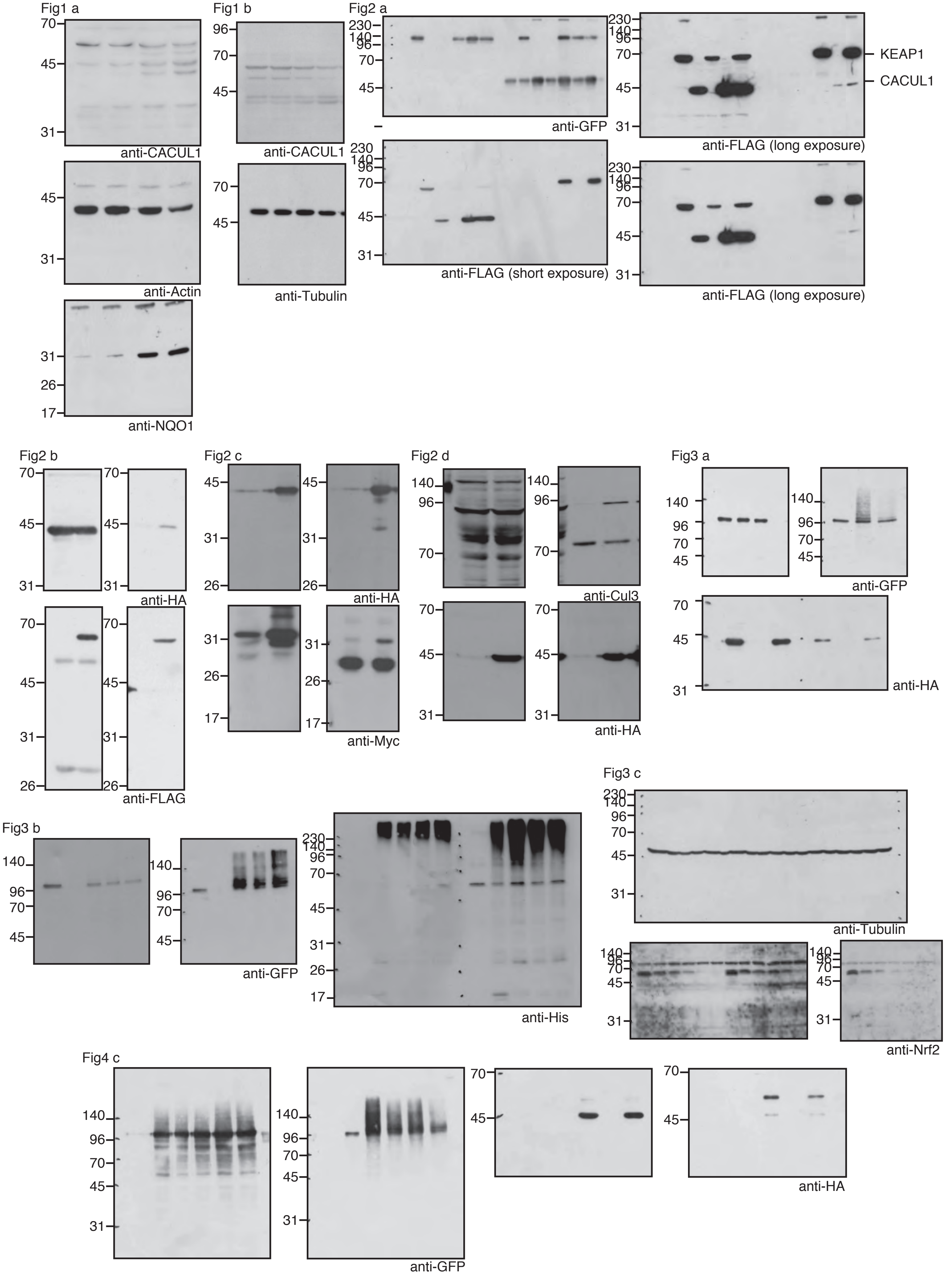

Supplementary Figure S7. Un-cropped images for western blots included in the main manuscript.
